# A Randomized Three-Arm Double-Blind Placebo-Controlled Study of Homeopathic Treatment of Children and Youth with Attention-Deficit/Hyperactivity Disorder

**DOI:** 10.1089/jicm.2023.0043

**Published:** 2024-03-15

**Authors:** David Brulé, Beth Landau-Halpern, Violeta Nastase, Marcia Zemans, Nicholas Mitsakakis, Heather Boon

**Affiliations:** ^1^ Leslie Dan Faculty of Pharmacy, University of Toronto, Toronto, Canada.; ^2^ Riverdale Homeopathic Clinic, Toronto, Canada.; ^3^ Centre for Addiction and Mental Health, Toronto, Canada.; ^4^ Department of Psychiatry, Temerty Faculty of Medicine, Psychiatry, University of Toronto, Toronto, Ontario, Canada.; ^5^ Children's Hospital of Eastern Ontario Research Institute, Ottawa, Canada.; ^6^ Dalla Lana School of Public Health, University of Toronto, Toronto, Canada.

**Keywords:** attention-deficit/hyperactivity disorder, homeopathy, complementary and alternative medicine, ADHD, pediatrics, mental health

## Abstract

**Objectives:**

Approximately 30% of children diagnosed with attention-deficit/hyperactivity disorder (ADHD), the most prevalent mental health disorder in children worldwide, do not respond to conventional pharmaceutical treatments. Previous studies of homeopathic treatment for ADHD have been inconclusive. The objectives of this study were to determine if there

(a) is an overall effect of homeopathic treatment (homeopathic medicines plus consultation) in the treatment of ADHD;
(b) are any specific effects the homeopathic consultation alone in the treatment of ADHD; and
(c) are any specific effects of homeopathic medicines in the treatment of ADHD.

**Design:**

The design was a randomized double-blind placebo-controlled clinical trial.

**Setting/Location:**

Toronto, Canada.

**Subjects:**

Children aged 6–16 years diagnosed with ADHD.

**Interventions:**

Participants were randomized to one of three arms: Arm 1 (Remedy and Consultation); Arm 2 (Placebo and Consultation); or Arm 3 (Usual Care).

**Outcome Measures:**

Primary Outcome was the change of CGI-P T score between baseline and 28 weeks.

**Results:**

There was an improvement in ADHD symptoms as measured by the Conner 3 Global Index-Parent *T*-score in the two groups (Arms 1 and 2) that received consultations with a homeopathic practitioner when compared with the usual care control group (Arm 3). Parents of the children in the study who received homeopathic consultations (Arms 1 and 2) also reported greater coping efficacy compared with those receiving usual care (Arm 3). There was no difference in adverse events among the three study arms.

**Conclusions:**

In this study, homeopathic consultations provided over 8 months with the use of homeopathic remedy was associated with a decrease in ADHD symptoms in children aging 6–16 years when compared with usual treatment alone. Children treated with homeopathic consultations and placebo experienced a similar decrease in ADHD symptoms; however, this finding did not reach statistical significance when correcting for multiple comparisons. Homeopathic remedies in and of themselves were not associated with any change in ADHD symptoms.

**Clinical Trial Registration:**

This trial was registered on ClinicalTrials.gov; NCT02086864.

## Introduction

Attention-deficit/hyperactivity disorder (ADHD) is a neurodegenerative disorder and the most prevalent mental disorder in children worldwide.^1^ Recent U.S. data suggest that 9.4% of children aging 2–17 years in the United States have been diagnosed with ADHD at one point in their childhood.^2^ Many children and adolescents find effective treatment with pharmaceutical medications and/or behavioral interventions; however, ∼30% of diagnosed children are deemed as nonresponders.^3^ Two-thirds of those with ADHD report to be using traditional, complementary, and alternative medicine treatments of various kinds.^4^

Homeopathy involves the treatment of patients using natural substances that are diluted and succussed (vigorous shaking).^5^ Results of systematic reviews show a slight positive effect of homeopathic *medicines* when compared with placebo; however, the mechanism of action is unknown and the paucity of studies make the conclusion tentative at best.^6^ Research on the effectiveness of homeopathic *treatment* (i.e., seeing a homeopathic practitioner) show more encouraging results.^7,8^

A review of clinical trials investigating homeopathic treatment for ADHD published in the Cochrane database in 2009 was inconclusive due to the heterogeneity of the trials.^9^ In their series of systematic reviews of different approaches to homeopathic treatment, Mathie et al.^10–12^ suggest that individualized homeopathy shows the most promise of possible efficacy in treating health conditions.^9,13–16^

Sixty-three percent of participants in the open-label pilot study (*n* = 36) of individualized homeopathy for ADHD experienced a clinically significant improvement in the primary outcome (Conners 3 Global Index-Parent [CGI-P] *T*-score).^17^ Given the open-label nature of the pilot study, it was not clear what accounted for the positive outcomes of many patients. The randomized controlled study reported here was designed to investigate that question.

This study was approved by the Centre for Addiction and Mental Health Research Ethics Board (REB No. 2013-079), the University of Toronto Health Sciences Research Ethics Board, and Health Canada.

## Design and Interventions

The objectives of this study were to determine if there

(a) is an overall effect of homeopathic treatment (homeopathic medicines plus consultation) in the treatment of ADHD;
(b) are any specific effects the homeopathic consultation alone in the treatment of ADHD; and
(c) are any specific effects of homeopathic medicines in the treatment of ADHD.

**FIG. 1.**
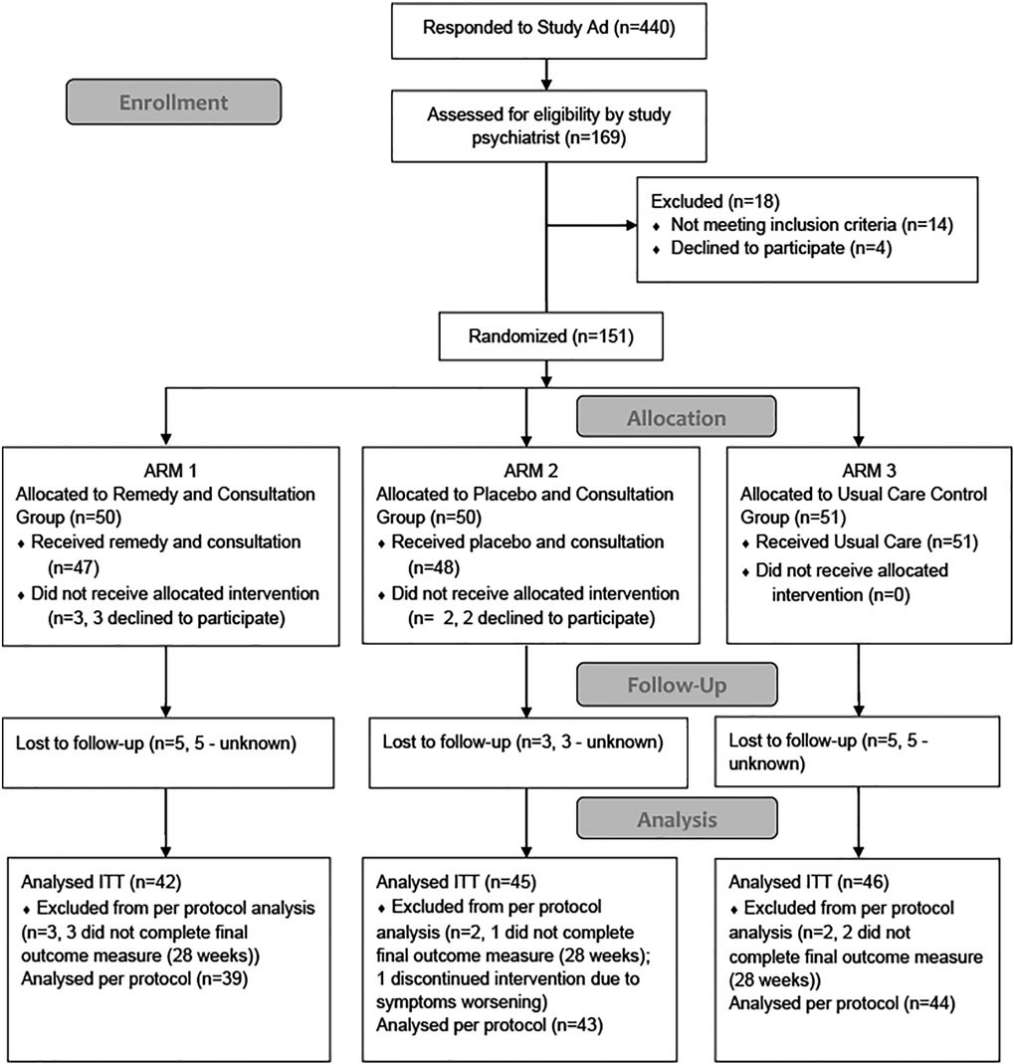
CONSORT flow diagram. ITT, intention to treat.

The design was a randomized double-blind placebo-controlled clinical trial (Fig. 1). Participants were randomized to one of three arms:

*Arm 1 (Remedy and Consultation):* a treatment arm where the participants received homeopathic consultation plus a homeopathic remedy prescribed by a classical homeopath licensed to practice in the Province of Ontario, Canada.

*Arm 2 (Placebo and Consultation):* a treatment arm where the participant received homeopathic consultation from a classical homeopath licensed to practice in the Province of Ontario, Canada plus a placebo remedy.

*Arm 3 (Usual Care):* a control group where the participant did not receive homeopathic treatment as part of the study but continued with their usual care.

Participants recruited from the community underwent a confirmatory diagnostic interview with the study psychiatrist.

### Participants

#### Inclusion and exclusion criteria

Inclusion criteria: (1) diagnosis of ADHD confirmed by study psychiatrist; (2) minimum baseline score on the Conners 3 scale that is 1.5 standard deviations above the population norm based on the age and sex of the participant; (3) ages 6–16 years; (4) able to ingest medications in liquid or in lactose/sucrose granule form; (5) if currently taking medication for ADHD, are on a stable dose for a minimum of 6 weeks before beginning the study; (6) have an estimated I.Q. within the normal range; and (7) have parents/guardians who are able to read and write in English or who are able to provide a certified English translator for the consenting process and provide an English translator of their choosing to all other study-related appointments.

Exclusion criteria: (1) diagnosis with an additional serious mental health disorder including, but not limited to, the following: conduct disorder, autism spectrum disorder, bipolar disorder, and major depressive disorder; (2) any significant suicidality; (3) an addiction to any substance; (4) taking any other prescription medication aside from a stable dose of ADHD medication; (5) a history of head injury (with sequelae), seizures, or organ system damage; or (6) pregnant or breastfeeding.

#### Patient recruitment

Participants from the community were recruited from July 2014 to February 2020 using a variety of methods (word of mouth, posters, seminars, ADHD advocacy group contact, referrals from a mental health hospital). Parents/guardians of the participants provided written informed consent; child participants provided initial and ongoing assent.

### Outcomes

The primary outcome was the change in *T*-score of the CGI-P, measured at the eighth visit compared with baseline. The CGI-P is a validated measure of 10 questions related to symptoms of ADHD embedded in the 100 question Conners 3 Parent questionnaire.^18^

There were five types of secondary outcome measures:

(a) Alternative Measures for ADHD Symptoms: Conners ADHD Index Probability score, Conners Content Scale *T*-scores for Inattention and Hyperactivity/Impulsivity, and Conners DSM-IV-TR Symptom Scale *T*-scores for the ADHD Inattentive and ADHD Hyperactive-Impulsive subtypes.^18^ In addition, the authors compared clinicians' perceptions of Arm 1 and Arm 2 participants' global improvement over the course of the study period using the 7-point clinician's Clinical Global Impression-Improvement (CGI-I) scale^19^ after visit 8 (week 28).
(b) Quality of Life: Change in quality of life from baseline to Visit 8 was assessed using the validated and reliable PedsQL™ measurement model (www. Pedsql.org).^20^ Three versions of the generic core scale (23 items) child self-report were used—one validated for children aging 5–7 years, one validated for children 8–12 years, and one validated for teens aging 13–18 years.^20^
  To assess any change in sense of mastery as well as ability to cope, the authors assessed the change from baseline to Visit 8 for the Mastery subscale of the “Resiliency Scales for Children & Adolescents-A Profile of Personal Strengths (RSCA)”,^21^ the Coping Efficacy Scale (parent and participant perception)^22^ and the Mastery subscale of the Control Beliefs Inventory.^23^
(c) At each homeopathic consultation, parents were specifically asked to report any suspected adverse events. All reported adverse events were categorized according to the Common Terminology Criteria for Adverse Events (CTCAEv4).^24^ Any severe adverse event (defined according to Health Canada as life-threatening, requiring hospitalization, or resulting in permanent disability) was referred to the study Data and Safety Monitoring Board for evaluation.

In addition to the outcome measures, demographic data were collected at baseline for all participants including gender, age, weight, concurrent medications (prescription, over the counter, and natural health products), and any changes in other forms of therapy (behavioral interventions, diet therapy, and other).

### Intervention

#### Homeopathic consultation (Arms 1 and 2)

Each initial 90–120 min classical homeopathic assessment was conducted by one of two homeopaths licensed in Ontario, Canada who have extensive clinical experience treating children with ADHD and involved a verbal interview with the study participant and a parent or guardian, assessing the child's physical and emotional symptoms as well as the history and course of the symptoms. Clinicians paid particular attention to signs and symptoms that were strange, rare, and peculiar when making their individual remedy choice decision.

Participants were formally assessed (by their homeopath) in person or by telephone approximately every 4 weeks. The practitioner was available by telephone between consultations. At any time during the study period the practitioner was able to make remedy or dosage changes if it was deemed to be in the best interest of the participant. The study treatment continued either until 28 weeks from intake or eight consultations (including intake consultation) whichever occurred first.

#### Homeopathic remedy and placebo (Arms 1 and 2)

The homeopathic practitioner prescribed a single individualized homeopathic remedy (study medication) for each patient based on Hahnemannian principles as outlined in the sixth edition on the Organon of Medical Art^5^ (maximum three times per day) from among the homeopathic medicines manufactured by Boiron Canada and currently approved for sale in Canada by Health Canada. Homeopathic potencies ranged from 6CH to 10M.

The homeopathic medicine prescription was forwarded to the study pharmacist who mailed either the manufactured homeopathic medicine or the placebo to patients.

Placebo medication was identical in taste and texture to the homeopathic medication and consisted of a 3.5 mm unmedicated lactose/sucrose granule. In the event of a water dosing prescription, the lactose/sucrose granule was mailed to the participant and the parent/guardian prepared the water dose remedy by dissolving three to four granules embedded with homeopathic medicine (or placebo) in 250 mL spring water.

#### Arm 3: usual care

Participants in the Usual Care arm of the study did not receive any homeopathic treatment (neither homeopathic remedy/placebo nor consultation) during the study period and completed all outcome measures on the same schedule as the other two groups. Participants and their caregivers could choose to undertake any other therapy (medication, behavioral interventions, etc.) as recommended by their regularly treating health care providers; however, such interventions were not prescribed as part of the study. All study participants could start or continue any treatment for their ADHD during the study.

### Randomization

Randomization was 1:1:1 for the three arms of the study in blocks of 6. An independent randomization service was contracted to perform the randomization.^25^

### Blinding

The entire study team was blinded to the allocation of participants in Arms 1 and 2. Arm 3 (Usual Care) control group allocation was known only by the study coordinator (D.B.) during the data collection phase. The statistician (N.M.), who conducted the primary data analysis, was blinded to group allocation for all three groups.

### Participant incentives

Each participant in Arms 1 and 2 received homeopathic consultations and medication or placebo throughout the duration of the trial free of charge. In addition, at the conclusion of the study, all participants in Arms 1 and 2 were given vouchers for three free homeopathic consultations. Arm 3 participants received vouchers for five free homeopathic consultations. These voucher consultations were not part of the study.

### Sample size

The sample size was estimated assuming that the comparisons between the three study Arms would be performed using the method of analysis of covariance (ANCOVA), adjusting for baseline score (and possibly other covariates). The “Reliable Change Index” for the CGI-P *T*-score is 7.36.^18^ The authors used this value as the desirable detectable difference between each of the three arms. Based on their pilot study, they estimated the correlation between the *T*-scores at baseline and after eight consultations to be ∼0.7.^17^ Accounting for multiplicity and assuming a dropout rate of 25%, a sample size of 59 patients per arm was identified as sufficient to achieve power equal to 0.8, under two-tailed significance level of 0.05.

### Statistical analysis

Linear models were used for the analysis of the primary outcome, the change of CGI-P *T*-score between baseline and 28 weeks measurements. In the primary analysis (complete case analysis), only study participants who completed questionnaires both at baseline and at visit 8 were included. The models were adjusted for the baseline CGI-P *T*-scores and presence/absence of ADHD medication at baseline. Following a procedure of fixed hierarchical order of comparisons,^26^ the authors first compared Arm 1 (Remedy and Consultation) and Arm 3 (Usual Care) as per the objective a. For this comparison, they used a significance level *α* = 0.05. Subsequently, as per objectives b and c, they made two comparisons between Arms 2 (Placebo and Consultation) and 3 (Usual Care) and between Arms 1 (Remedy and Consultation) and 2 (Placebo and Consultation). After multiple testing correction, they used significance level *α* = 0.05/2 = 0.025 for these two comparisons.

In addition to the complete case analysis, the authors performed a secondary sensitivity analysis, where missing data were imputed using multiple imputation and chained equations.^27^ Five imputed data sets were generated and linear models similar to those used in the primary analysis were applied, and subsequently the results over the five models were pooled.^28^ Since this analysis uses *all* randomized participants, it fulfills the role of an *intention to treat* analysis.

Secondary outcomes were analyzed similarly to the primary analysis.

A *post hoc* analysis was completed to compare the three groups with respect to the proportion of participants who achieved a clinically significant improvement in their symptoms using the Reliable Change Index—a measure of clinical relevance built into the Conners 3 questionnaire. According to the Conners Manual,^18^ the Reliable Change Index (7.36) is the minimum change on the CGI-P *T*-score from baseline to endpoint that has been found to be clinically relevant. Outcomes based on Reliable Change Index were compared without adjustment, using chi-squared test as well as, adjusting for baseline CGI-P *T*-score and ADHD medications, using a logistic regression model and pairwise comparisons. *p*-Values were not adjusted for multiplicity.

This trial was registered on ClinicalTrials.gov; NCT02086864 (https://www.clinicaltrials.gov).

## Results

### Participants

A total of 151 participants gave informed consent and were randomized; 5 participants withdrew before any intervention (Fig. 1).

As shown in Table 1, there were no significant differences in the baseline characteristics between the three groups.

**Table 1.** Baseline Demographic and Clinical Characteristics of Study Participants

Characteristic	Arm 1 Remedy and Consultation (*n* = 47)	Arm 2 Placebo and Consultation (*n* = 48)	Arm 3 Usual Care Control (*n* = 51)	
	Mean ± SD or* n *(%)	Mean ± SD or* n *(%)	Mean ± SD or* n *(%)	*p*
Age	9.89 ± 2.85	9.60 ± 2.68	9.55 ± 2.90	0.812
Male	33 (70.2)	39 (81.3)	35 (68.6)	0.285
Taking conventional medications for ADHD	12 (25.5)	12 (25)	10 (19.6)	0.746
Taking natural health products for ADHD	14 (29.8)	18 (37.5)	14 (27.5)	0.540
Conners 3 Global Index-Parent *T*-Score	79.23 ± 9.98	77.96 ± 12.29	80.20 ± 9.82	0.587
PedsQL	69.79 ± 15.80 (*n* = 42)	69.58 ± 13.39 (*n* = 44)	70.00 ± 17.80 (*n* = 44)	0.992
PedsQL–Physical	80.36 ± 16.78 (*n* = 42)	79.15 ± 13.94 (*n* = 44)	79.40 ± 19.49 (*n* = 44)	0.941
Resiliency Scales for Children and Adolescents-Mastery subscale	2.63 ± 0.45 (*n* = 26)	2.81 ± 0.53 (*n* = 27)	2.77 ± 0.56 (*n* = 22)	0.434
Coping Efficacy Scale-Parent	2.87 ± 0.91 (*n* = 43)	2.51 ± 0.95 (*n* = 43)	2.65 ± 1.03 (*n* = 41)	0.201

ADHD, attention-deficit/hyperactivity disorder; SD, standard deviation.

For a complete list homeopathic medicines prescribed, see Supplementary Table S1.

### Impact on children's ADHD symptoms

Participants in both Arm 1 (Remedy and Consultation) as well as Arm 2 (Placebo and Consultation) had a significant change in CGI-P *T*-score (representing symptom improvement) over time from baseline to 28 weeks, whereas those in Arm 3 (Usual Care) did not have a significant change over time (Table 2).

**Table 2.** Mean Pre–Post Conners 3 Global Index-Parent *T*-Score for the Three Treatment Groups

Group	Initial* T*-score	Final* T*-score	Change in* T*-score*^a^*	*p* ^b^
Arm 1 Remedy and Consultation (*n* = 39)	78.64	72.41	−6.23 (1.50)	0.024
Arm 2 Placebo and Consultation (*n* = 43)	78.23	71.86	−6.37 (1.37)	0.029
Arm 3 Usual Care Control (*n* = 44)	80.18	78.00	−2.18 (1.22)	0.352

^a^ Standard error is also shown.

^b^ Paired samples *t*-test.

As can be seen in Table 3, the results indicate a statistically significant difference between Arm 1 (Remedy and Consultation) and Arm 3 (Usual Care) (*p* = 0.037). The authors did not find a significant difference between Arm 3 and Arm 2 (Placebo and Consultation) at the 0.025 level (as per the multiple comparison adjustment procedure described in the Statistical Analysis section), although the *p*-value (0.026) was approaching statistical significance. They found no evidence for difference between Arm 1 and Arm 2.

**Table 3.** Linear Models Analysis of CGI-P *T*-Score Across Three Treatment Groups Adjusting for Medications and Baseline Measurements

Group comparisons	Estimate	Std. error	*t*	Pr(>|*t*|)
Arm 3 (Usual Care Control Group) vs. Arm 1 (Remedy and Consultation Group)	4.1245	1.9545	2.110	0.0369
Arm 3 (Usual Care Control Group) vs. Arm 2 (Placebo and Consultation)	4.2981	1.9079	2.253	0.0261
Arm 2 (Placebo and Consultation) vs. Arm 1 (Remedy and Consultation Group)	−0.1735	1.9578	−0.089	0.9295

Sensitivity analysis using multiply imputed missing values for all randomized patients (playing the role of an intention to treat analysis) was consistent with the primary analysis. Pooled estimates from a linear model comparing CGI-P *T*-score change identified a mean difference of 3.652 U (*p* = 0.062) between Arm 3 and Arm 1, and a mean difference of 3.673 U (*p* = 0.046) between Arm 3 and Arm 2.

### Secondary analyses

Most secondary outcome measures were not significant at the 0.05 level. There were no significant differences among the majority of the secondary measures (Supplementary Tables S2 and S3).

There were no significant differences in change of the Total Score or the Psychological subscore of the PedsQL (quality of life scale) among any of the three arms. However, those in Arm 1 (Remedy and Consultation) had a larger positive change on the PedsQL Physical Score, compared with those in Arm 3 (Usual Care) (*p* = 0.038) (Supplementary Table S4).

There was a significant difference among the three study groups with respect to the Coping Efficacy Scale, which was completed by the parent and is a measure of how the parent feels the child is coping with their day-to-day ADHD symptoms. As can be seen in Table 4, both Arm 1 (Remedy and Consultation) as well as Arm 2 (Placebo and Consultation) demonstrated a positive change in coping efficacy when compared with Arm 3 (Usual Care). There was no significant difference between the Arm 1 and Arm 2. Sociodemographic factors (i.e., age, conventional medication use at intake, gender, and initial ADHD score [CGI-P *T*-score]) were not associated with the change in parent's coping efficacy score; however, the coping efficacy score was associated with change in CGI-P *T*-score (linear regression analysis; *p* < 0.001, adjusted *r*^2^ = 0.159).

**Table 4.** Linear Models Analysis of Parents' Coping Efficacy Score Across Three Treatment Groups

Group comparisons	Estimate	Std. error	*t*	Pr(>|*t*|)
Arm 3 (Usual Care Control Group; *n* = 31) vs. Arm 1 (Remedy and Consultation Group; *n* = 35)	−0.545	0.212	−2.566	0.012
Arm 3 (Usual Care Control Group; *n* = 31) vs. Arm 2 (Placebo and Consultation; *n* = 39)	−0.641	0.207	−3.092	0.003
Arm 2 (Placebo and Consultation; *n* = 39) vs. Arm 1 (Remedy and Consultation Group; *n* = 35)	0.096	0.202	0.475	0.636

### *Post hoc* analyses

Results of the reliable change *post hoc* analysis indicate that there is an evident difference between Arms 3 (Usual Care) and 1 (Remedy and Consultation), as well as between Arms 3 and 2 (Placebo and Consultation), but not between Arms 2 and 1 (Table 5). Pairwise comparisons following a logistic regression model, adjusted for baseline CGI-P *T*-score and ADHD medication, indicated a more prevalent outcome in Arm 1 in comparison with Arm 3 (*p* = 0.023), and in Arm 2 in comparison with Arm 3 (*p* = 0.003). No significant difference was found between Arm 2 and Arm 1 (*p* = 0.445). *p*-Values were not adjusted for multiple comparisons.

**Table 5.** Descriptive Analysis of Reliable Change Outcome

	Arm 1 Remedy and Consultation (*n* = 39)	Arm 2 Placebo and Consultation (*n* = 43)	Arm 3 Usual Care Control (*n* = 44)	*p* ^a^	Missing
CGI-P_T Reliable Change Index Met	14 (35.9%)	19 (44.2%)	6 (13.6%)	0.006	16.6

^a^ Chi-squared test.

CGI-P, Conners 3 Global Index-Parent.

### Adverse effects

There was no significant difference in the number of adverse events reported across the three arms (Supplementary Table S5). All adverse effects reported in all three groups were Grade 1 using the CTCAEv4 criteria with the most common being increased hyperactivity, emotional outbursts, and increased tics. See Supplementary Table S5 for list and frequency of adverse events possibly or probably related to the therapy.

## Discussion

This study found an improvement in ADHD symptoms as measured by the CGI-P *T*-score in the two groups (Arm 1 and Arm 2) that received consultations with a homeopathic practitioner when compared with the usual care control group. It should be noted that the authors were not able to blind participants to the homeopathic consultation and, therefore, it is possible that these effects were nonspecific/placebo/expectation effects.

There were two secondary measures that were statistically significant—the parent/guardian report of coping efficacy and the PedsQL physical subscale. The difference in the coping measure suggests that having a homeopathic consultation may lead to improvement in coping as perceived by the parent/guardian, whereas the PedsQL (physical) difference suggests that homeopathic consultations with homeopathic remedy may result in a better physical quality of life. Since parents were completing the primary ADHD outcome measure, it is possible that their perception of how their child was coping influenced their perceptions of the severity of their child's symptoms. Previous research exploring the possibility of a link between coping efficacy and perception of symptoms is mixed.^29,30^

The findings are generally consistent with a recent meta-analysis that concluded that “(i)ndividualized homeopathy showed a clinically relevant and statistically robust effect in the treatment of ADHD.”^31^ Similar to the meta-analysis, the authors found individualized homeopathy (consultation plus remedy) resulted in improvement in ADHD symptoms. However, the data suggest that this effect is not due to the remedy component of the intervention.

A key strength of this study was its design, which maximized model validity.^14^ This research studied classical homeopathic treatment as it is normally practiced including giving study clinicians the ability to select individualized remedies, revise remedy choices throughout the study, and allowed sufficient time to see effects (if any) of the treatment(s) and allowing conventional medication treatment. This design enhances the applicability of the findings to real-world clinical practice. The one limit on the clinicians' practice was that they were required to only prescribe homeopathic medicines available over the counter in Canada as part of this study. In contrast, in their normal daily practice, they can prescribe any homeopathic medicine available globally.

A second important limitation to this study is that the authors were not able to recruit the planned number of participants due to the onset of the COVID-19 pandemic and the subsequent halt of all clinical trials in the jurisdiction. Thus, the power of the analyses to detect differences between groups was slightly less than planned.

## Conclusion

In this study, homeopathic consultations provided over 8 months with the use of homeopathic remedy was associated with a decrease in ADHD symptoms in children aging 6–16 years when compared with usual treatment alone. Children treated with homeopathic consultations and placebo experienced a similar decrease in ADHD symptoms; however, this finding did not reach statistical significance when correcting for multiple comparisons. Homeopathic remedies in and of themselves were not associated with any change in ADHD symptoms. Parents of the children in the study who received homeopathic consultations (with or without homeopathic remedy) also reported greater coping efficacy.

Further investigation with respect to the mechanism of action of this intervention, if/how the homeopathic remedy is related to the outcome and/or if other types of consultations or psychological interventions may also be beneficial is warranted.

## Supplementary Material

Supplemental data

Supplemental data

Supplemental data

Supplemental data

Supplemental data

## Data Sharing

Raw data may be available from the authors upon reasonable request.
