# Supplementary material for: A Randomized Three-Arm Double-Blind Placebo-Controlled Study of Homeopathic Treatment of Children and Youth with Attention-Deficit/Hyperactivity Disorder
Source: J Integr Complement Med. 2024 Mar 15;30(3):279–87. doi: 10.1089/jicm.2023.0043 (PMC10960167; doi:10.1089/jicm.2023.0043)
Supplement: Supplemental data [file Suppl_TableS1.docx]

**Supplementary Table 1: List of Homeopathic Remedies Prescribed**

| **Remedy name** | **Frequency used^a^** |  | **Remedy name** | **Frequency used^a^** |
| --- | --- | --- | --- | --- |
| Hyoscyamus Niger | 21 |  | Gallicum Acid | 2 |
| Phosphorus | 17 |  | Magnesium Muriaticum | 2 |
| Lycopodium Clavatum | 12 |  | Natrum Carbonicum | 2 |
| Tarentula Hispanica | 11 |  | Spongia Tosta | 2 |
| Sulfur | 10 |  | Apis Mellifica | 1 |
| Lachesis Mutus | 8 |  | Argentum Metallicum | 1 |
| Silicea | 7 |  | Aurum Metalicum | 1 |
| Tuberculinum | 7 |  | Baryta Phosphorica | 1 |
| Belladonna | 6 |  | Bufo Rana | 1 |
| Veratrum Album | 6 |  | Calcarea Bromatum | 1 |
| Pulsatilla | 5 |  | Calcarea Carbonica | 1 |
| Anacardium Orientale | 4 |  | Colchicum Autumnale | 1 |
| Aranea Ixobola | 4 |  | Crocus Sativus | 1 |
| Nux Vomica | 4 |  | Gelsemium Sempervirens | 1 |
| Sepia | 4 |  | Ignatia Amara | 1 |
| Baryta Carbonica | 3 |  | Kalium Carbonicum | 1 |
| Medorrhinum | 3 |  | Kalium Phosphoricum | 1 |
| Natum Phosphoricum | 3 |  | Kalium Sulphuricum | 1 |
| Staphysagria | 3 |  | Menyanthes Trifoliata | 1 |
| Stramonium | 3 |  | Mercurius Solubilis | 1 |
| Argentum Nitricum | 2 |  | Picricum Acid | 1 |
| Calcarea Phosphorica | 2 |  | Saccharum Officinale | 1 |
| Causticum | 2 |  | Thuja Occidentalis | 1 |
| Chamomilla | 2 |  |  |  |
