# Supplementary material for: A Randomized Three-Arm Double-Blind Placebo-Controlled Study of Homeopathic Treatment of Children and Youth with Attention-Deficit/Hyperactivity Disorder
Source: J Integr Complement Med. 2024 Mar 15;30(3):279–87. doi: 10.1089/jicm.2023.0043 (PMC10960167; doi:10.1089/jicm.2023.0043)
Supplement: Supplemental data [file Suppl_TableS2.docx]

**Supplementary Table 2.1: Change from baseline in Conners ADHD Index Probability T-score**

| **Group comparisons** | **Estimate** | **Std. Error** | **t value** | **Pr(>\|t\|)** |
| --- | --- | --- | --- | --- |
| Arm 3 (Usual Care Control Group; n=44) vs  Arm 1 (Remedy & Consultation Group; n=39) | 7.281 | 4.222 | 1.724 | 0.087 |
| Arm 3 (Usual Care Control Group; n=44) vs  Arm 2 (Placebo & Consultation; n=43) | 5.013 | 4.133 | 1.213 | 0.228 |
| Arm 2 (Placebo & Consultation; n=43) vs Arm 1 (Remedy & Consultation Group; n=39) | 2.268 | 4.233 | 0.536 | 0.593 |

**Supplementary Table 2.2: Change from baseline in Conners Content Scale for Inattention T-score**

| **Group comparisons** | **Estimate** | **Std. Error** | **t value** | **Pr(>\|t\|)** |
| --- | --- | --- | --- | --- |
| Arm 3 (Usual Care Control Group; n=44) vs  Arm 1 (Remedy & Consultation Group; n=39) | 2.545 | 2.040 | 1.248 | 0.215 |
| Arm 3 (Usual Care Control Group; n=44) vs  Arm 2 (Placebo & Consultation; n=42) | 1.996 | 1.997 | 0.999 | 0.320 |
| Arm 2 (Placebo & Consultation; n=42) vs Arm 1 (Remedy & Consultation Group; n= 39) | 0.549 | 2.059 | 0.267 | 0.790 |

**Supplementary Table 2.3: Change from baseline in Conners Content Scale for Hyperactivity/Impulsivity T-score**

| **Group comparisons** | **Estimate** | **Std. Error** | **t value** | **Pr(>\|t\|)** |
| --- | --- | --- | --- | --- |
| Arm 3 (Usual Care Control Group; n=44) vs  Arm 1 (Remedy & Consultation Group; n=39) | 3.995 | 2.109 | 1.894 | 0.061 |
| Arm 3 (Usual Care Control Group; n=44) vs  Arm 2 (Placebo & Consultation; n=43) | 3.165 | 2.061 | 1.536 | 0.127 |
| Arm 2 (Placebo & Consultation; n=43) vs Arm 1 (Remedy & Consultation Group; n=39) | 0.830 | 2.107 | 0.394 | 0.694 |

**Supplementary Table 2.4: Change from baseline in Conners DSM-IV-TR Symptom Scale for the ADHD Inattentive subtype T-score**

| **Group comparisons** | **Estimate** | **Std. Error** | **t value** | **Pr(>\|t\|)** |
| --- | --- | --- | --- | --- |
| Arm 3 (Usual Care Control Group; n=43) vs  Arm 1 (Remedy & Consultation Group; n=39) | 1.742 | 2.212 | 0.788 | 0.432 |
| Arm 3 (Usual Care Control Group; n=43) vs  Arm 2 (Placebo & Consultation; n=43) | 2.011 | 2.159 | 0.931 | 0.354 |
| Arm 2 (Placebo & Consultation; n=43) vs Arm 1 (Remedy & Consultation Group; n=39) | -0.268 | 2.213 | -0.121 | 0.904 |

**Supplementary Table 2.5: Change from baseline in Conners DSM-IV-TR Symptom Scale for the ADHD Hyperactive-Impulsive subtype T-score**

| **Group comparisons** | **Estimate** | **Std. Error** | **t value** | **Pr(>\|t\|)** |
| --- | --- | --- | --- | --- |
| Arm 3 (Usual Care Control Group; n=44) vs  Arm 1 (Remedy & Consultation Group; n=39) | 4.170 | 2.148 | 1.941 | 0.055 |
| Arm 3 (Usual Care Control Group; n=44) vs  Arm 2 (Placebo & Consultation; n=43) | 4.101 | 2.096 | 1.957 | 0.053 |
| Arm 2 (Placebo & Consultation; n=43) vs Arm 1 (Remedy & Consultation Group; n=39) | 0.069 | 2.148 | 0.032 | 0.974 |
