# Supplementary material for: A Randomized Three-Arm Double-Blind Placebo-Controlled Study of Homeopathic Treatment of Children and Youth with Attention-Deficit/Hyperactivity Disorder
Source: J Integr Complement Med. 2024 Mar 15;30(3):279–87. doi: 10.1089/jicm.2023.0043 (PMC10960167; doi:10.1089/jicm.2023.0043)
Supplement: Supplemental data [file Suppl_TableS3.docx]

**Supplementary Table 3: Clinical Global Impression-Improvement scale (CGI-I)**

|  | **Arm 1 (Remedy & Consultation Group) n=36** | **Arm 2 (Placebo & Consultation) n=35** |  |  |
| --- | --- | --- | --- | --- |
|  | **Mean (SE)** | **Mean (SE)** | **Difference (95%CI)^a^** | **Pr(>\|t\|)** |
| CGI-I | 2.47 (0.81) | 2.66 (0.80) | -0.19 (-0.57, 0.20) | 0.337 |

^a^95% Confidence Interval and p-value are obtained using t-test of 2 independent samples.
