# Supplementary material for: A Randomized Three-Arm Double-Blind Placebo-Controlled Study of Homeopathic Treatment of Children and Youth with Attention-Deficit/Hyperactivity Disorder
Source: J Integr Complement Med. 2024 Mar 15;30(3):279–87. doi: 10.1089/jicm.2023.0043 (PMC10960167; doi:10.1089/jicm.2023.0043)
Supplement: Supplemental data [file Suppl_TableS4.docx]

**Supplementary Table 4.1: Change from baseline in the Pediatric Quality of Life Inventory Physical sub-scale**

| **Group comparisons** | **Estimate** | **Std. Error** | **t value** | **Pr(>\|t\|)** |
| --- | --- | --- | --- | --- |
| Arm 3 (Usual Care Control Group; n=33) vs  Arm 1 (Remedy & Consultation Group; n=30) | 6.610 | 3.134 | 2.109 | 0.038 |
| Arm 3 (Usual Care Control Group; n=33) vs  Arm 2 (Placebo & Consultation; n=39) | 2.654 | 2.939 | 0.903 | 0.369 |
| Arm 2 (Placebo & Consultation; n=39) vs Arm 1 (Remedy & Consultation Group; n=30) | 3.956 | 3.018 | 1.311 | 0.193 |

**Supplementary Table 4.2: Change from baseline in the Pediatric Quality of Life Inventory Psychological sub-scale**

| **Group comparisons** | **Estimate** | **Std. Error** | **t value** | **Pr(>\|t\|)** |
| --- | --- | --- | --- | --- |
| Arm 3 (Usual Care Control Group; n=33) vs  Arm 1 (Remedy & Consultation Group; n=30) | 4.131 | 3.355 | 1.231 | 0.221 |
| Arm 3 (Usual Care Control Group; n=33) vs  Arm 2 (Placebo & Consultation; n=39) | 1.088 | 3.146 | 0.346 | 0.730 |
| Arm 2 (Placebo & Consultation; n=39) vs Arm 1 (Remedy & Consultation Group; n=30) | 3.043 | 3.230 | 0.942 | 0.348 |

**Supplementary Table 4.3: Change from baseline in the Pediatric Quality of Life Inventory Overall**

| **Group comparisons** | **Estimate** | **Std. Error** | **t value** | **Pr(>\|t\|)** |
| --- | --- | --- | --- | --- |
| Arm 3 (Usual Care Control Group; n=33) vs  Arm 1 (Remedy & Consultation Group; n=30) | 4.706 | 2.792 | 1.685 | 0.095 |
| Arm 3 (Usual Care Control Group; n=33) vs  Arm 2 (Placebo & Consultation; n=39) | 1.784 | 2.618 | 0.681 | 0.497 |
| Arm 2 (Placebo & Consultation; n=39) vs Arm 1 (Remedy & Consultation Group; n=30) | 2.922 | 2.688 | 1.087 | 0.280 |
