# Supplementary material for: A Randomized Three-Arm Double-Blind Placebo-Controlled Study of Homeopathic Treatment of Children and Youth with Attention-Deficit/Hyperactivity Disorder
Source: J Integr Complement Med. 2024 Mar 15;30(3):279–87. doi: 10.1089/jicm.2023.0043 (PMC10960167; doi:10.1089/jicm.2023.0043)
Supplement: Supplemental data [file Suppl_TableS5.docx]

**Supplementary Table 5.1: Number of adverse events reported per study arm**

|  | **Arm 1**  **Remedy & Consultation (n=47)** | **Arm 2**  **Placebo & Consultation (n=48)** | **Arm 3**  **Usual Care Control (n=51)** | **p-value** |
| --- | --- | --- | --- | --- |
| Adverse events reported^a^ (mean (SD)) | 1.26 (1.29) | 1.46 (1.85) | 1.10 (1.10) | 0.463 |

^a^adverse event graded as “unlikely”, “possibly” or “probably” related to the therapy

**Supplementary Table 5.2: Qualitative description of adverse events reported verum group and placebo group**

| **Adverse Event reported – Verum Group^a^** | **Number of times reported** |
| --- | --- |
| anger/fighting/disruptive | 7 |
| tics/increased tics | 6 |
| headaches | 2 |
| acute cold | 2 |
| irritability/emotional sensitivity | 2 |
| Rash | 1 |
| restless | 1 |
| appetite loss | 1 |
| bedwetting | 1 |
| pinworms | 1 |
| eczema increased | 1 |
| sore throat | 1 |
| stomach ache | 1 |
| **Adverse Event reported – Placebo Group^a^** | **Number of times reported** |
| irritability/emotional sensitivity | 7 |
| hyperactivity | 5 |
| anger/fighting/disruptive | 5 |
| loquacity | 1 |
| hand and feet numbness | 1 |

^a^adverse event graded as “possibly” or “probably” related to the therapy
